# Rare, spontaneous trans-splenic shunt and intra-splenic collaterals with attendant splenic artery aneurysms in an adult patient with compensated cirrhosis and portal hypertension

**DOI:** 10.1093/gastro/gou047

**Published:** 2014-07-09

**Authors:** Cyriac Abby Philips, Lovkesh Anand, K.N. Chandan Kumar, Vivek Kasana, Ankur Arora

**Affiliations:** ^1^Department of Hepatology, Institute of Liver and Biliary Sciences, New Delhi, India and ^2^Department of Radiology, Institute of Liver and Biliary Sciences, New Delhi, India

**Keywords:** cirrhosis, portal hypertension, portosystemic shunts, collateral pathways, splenomegaly

## Abstract

We present a rare case of spontaneous trans-splenic shunt and intra-splenic collaterals in a patient with liver cirrhosis and portal hypertension. The shunt and presence of cirrhosis and portal hypertension was incidentally detected by abdominal computed tomographic imaging during evaluation for abdominal pain. There has been a single report on the presence of trans-splenic shunt in two children with extra-hepatic portal venous obstruction but no cases that report intra-splenic collaterals: to the best of our knowledge, this is the first reported case of spontaneous trans-splenic shunt in the presence of intra-splenic collaterals and incidental multiple splenic artery aneurysms that developed in an adult with compensated cirrhosis and portal hypertension.

## INTRODUCTION

Johns and Evans in 1962 first described portosystemic collateral veins (PSCV) in portal hypertension [1]. Portosystemic collateral veins commonly occur in patients with both cirrhosis and portal hypertension (PHTN). PHTN leads to back-pressure transmission, which leads to increased flow in normal, hepatofugal flow-directed patent PSCV [2]. Large collateral veins form spontaneous shunts in such patients. In cirrhosis, the obstructed blood flow through vascular channels in the distorted parenchyma leads to re-opening of collapsed embryonic channels or reversal of flow within existing adult veins. This helps in by-passing the areas of high resistance [3, 4]. Any abdominal vein can become a potential collateral channel to the systemic circulation. The diagnosis of PHTN can be confirmed with a sensitivity of 70–80% in the presence of abdominal collateral veins.

Earlier it was believed that, the more severe and prolonged the PHTN, the higher the number of portosystemic shunt pathways. Evidence currently points to angiogenesis driven by vascular endothelial growth factor, which leads to more shunt formation in cirrhosis patients [5]. Current medical literature is rich with detailed mapping of portosystemic pathways in cirrhosis and the clinical implications, complications and therapeutic modalities involved in their management. Precise mapping of PSCV is important for therapeutic decisions and multi-detector computed tomography (MDCT) has commonly been used for this purpose. The areas of normal portosystemic anastomoses include:

- Left gastric veins, along with oesophageal veins draining into azygous vein.
- Superior rectal vein anastomosing with middle and inferior rectal veins (tributaries of internal iliac and pudental veins respectively).
- Paraumbilical and subcutaneous veins in the anterior abdominal wall.
- Tributaries of splenic and pancreatic veins anastomosing left renal vein the retroperitoneal space.
- Short veins between splenic and colic veins to lumbar veins of posterior abdominal wall and also veins in the bare area of liver (communicates with those of diaphragm and right internal thoracic vein)

In PHTN, dilation of these channels promotes development of varices at various sites in the body, mostly classified into two groups, the oesophago-gastric varices and ectopic varices. These varices are fed in the long term by spontaneously developing large shunts in the abdomen [6, 7]. The gastrorenal shunt is formed mainly by the lower branch of the inferior phrenic vein, which opens directly into the renal vein (spleno-gastro-phreno-renal shunt) or through left adrenal vein. The gastrocaval shunt drains through the upper branch of the inferior phrenic vein into the vena cava and is mostly continuous with the phrenicopericardial vein, which ultimately drains into the brachiocephalic vein. Direct splenorenal shunts provide communication between the splenic vein and the left renal vein, sometimes through the splenic capsule. This direct portosytemic shunting is similar to direct shunting of blood from the posterior branch of the left gastric vein to the para-oesophageal veins and azygous vein, without formation of oesophageal varices. Sometimes a direct shunt can be seen between the spleen and adrenal vein, bypassing the gastric area, called spleno-adrenalo-renal shunt [8, 9]. Spontaneous, indirect splenorenal shunts—characterized by the presence of a complete neurovascular pedicle traversing the gastrophrenic ligament—are rare. In such a scenario, the gastric collateral vein is connected to the left renal vein through the inferior phrenic vein and the middle capsular vein, this being known as gastro-phreno-capsulo-renal shunt.

Rare spontaneous shunts are seldom reported in the literature. In our case report, we have highlighted the presence of such a rarity—a trans-splenic shunt in association with rarer intra-splenic shunts—which has never been reported in compensated adult cirrhosis and portal hypertension. Our patient also had a large spontaneous splenorenal shunt with large splenic artery aneurysms against the background of high portal pressures.

## CASE REPORT

A 40-year-old woman presented to our outpatient department with complaints of progressive lethargy, loss of appetite and early satiety, associated with progressive left upper abdominal fullness over a period of four months. She denied the presence of jaundice, bleeding diathesis, abdominal distension or fever with prodrome. The patient was a teetotaller with no known comorbidities and neither she nor her family members had had previous liver diseases. On examination, the patient was found to be alert and oriented to time, place and person. Pallor was evident without icterus, cyanosis, lymphadenopathy or peripheral oedema. An abdominal examination revealed a liver that was palpable three finger breadths below the right costal margin, which was firm in consistency with sharp irregular margins with rough surface and without attendant bruit. There was massive tender splenomegaly without bruit. There was no evidence of free fluid in the abdomen and engorged and tortuous veins were absent over the anterior and posterior abdominal walls. The rest of the physical examination, covering cardiovascular, respiratory and central nervous systems, was non-contributory. Investigation revealed the presence of severe pancytopenia with haemoglobin of 7.2 g/dL, total leucocyte count of 1200 per mm^3^ with absolute neutrophil count of 750 cells per mm^3^ and platelet counts of 56 000 per mm^3^. A liver function test was normal, with only mild elevation in serum alkaline phosphatase. Tests for hepatitis B, hepatitis C, other viruses and ANA, ASMA, AMA, anti LKM-1 and IgG were negative. Tests of thyroid function and glycemic status were found to be normal.

**Figure 1:**
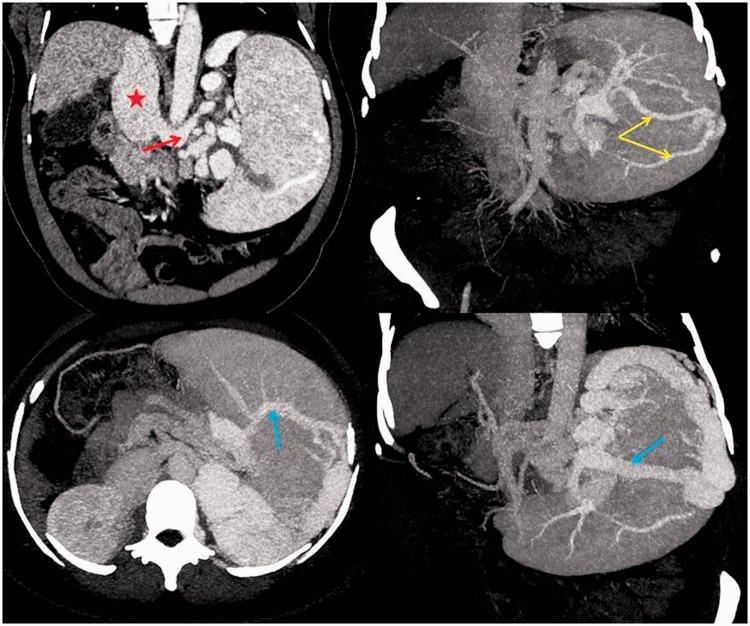
Computed tomography imaging of the abdomen with 3D reconstruction. Clockwise from left: splenorenal shunt from origin of the IVC (red star); left renal vein (red arrow) joining the splenic vein at the hilum; multiple intrasplenic collaterals (yellow arrows); large transplenic shunt (blue arrow); 3D sectional imaging of trans-splenic shunt (blue arrow) with intra-splenic collaterals.

**Figure 2:**
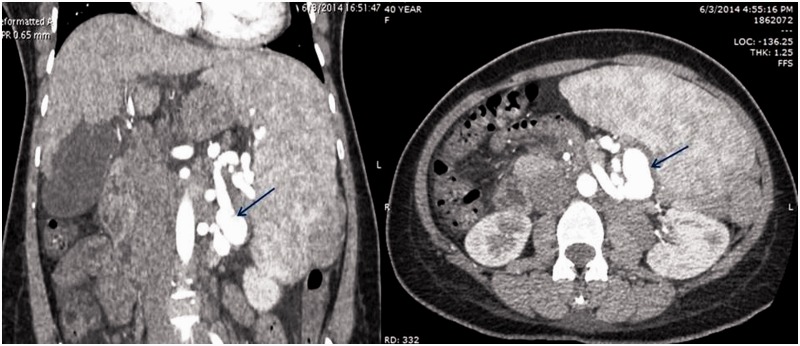
Computed tomography of the abdomen. Left to right: the presence of multiple splenic artery aneurysms in association with portosystemic collaterals (blue arrow).

Baseline ultrasound imaging of the abdomen was done, revealing the presence of cirrhotic architecture of the liver, with multiple abdominal collaterals and massive splenomegaly without evidence of ascites. The patient subsequently underwent an MDCT of the abdomen, which revealed that the liver was shrunken, with a span of approximately 9.8 cm craniocaudally, showing slight widening of the interlobar fissure with subtle undulating contour. The left lobe was relatively hypertrophied. There was no evidence of focal arterial-phase enhancing lesions. The main, right and left portal veins were markedly attenuated and their diameters were approximately 6, 5 and 5 mm, respectively. Splenic vein and superior mesenteric veins were dilated, at 12 and 11 mm, respectively, showing normal contrast opacification. There was a large tuft of tortuous and dilated perisplenic, retro-splenic and intra-splenic collaterals, deriving their afferent supply from the short gastric vein and draining through a giant spleno-renal shunt into the left renal vein. In addition, they were seen to drain into the systemic circulation through the left paravertebral venous plexus in the presence of paraoesophageal, perigastric, mesenteric and retroperitoneal collaterals. The spleen was massively enlarged, spanning 21 cm, with small areas of wedge infarcts and showing a large tuft of tortuous and dilated perisplenic, transplenic as well as intra-splenic collaterals. The splenic artery was dilated and tortuous with multiple flow-related aneurysms, the largest measuring up to 3 cm in diameter (Figures 1 and 2). Further, a trans-jugular liver biopsy was done with measurement of hepatic venous pressure gradient (HVPG) in the same sitting. The biopsy showed features of cirrhosis and the HVPG was 22 mm Hg.

An eventual diagnosis was made of portal hypertension with large varices, secondary to cryptogenic cirrhosis of the liver, and the patient was managed with adequate analgesia and bed rest for splenic infarct-related abdominal pain and beta blocker therapy as primary prophylaxis for upper gastrointestinal bleeds. She was discharged on the fourth day following admission and has since been on follow-up, to look for symptomatic hypersplenism or bleeding diathesis.

## DISCUSSION

PSCVs develop mainly in patient who have PHTN with or without cirrhosis, in order to decompress the splanchnic flow. PSCVs can be divided into congenital or spontaneous shunts and anatomically into intra-hepatic (connections between intra-hepatic portal vein and hepatic veins) and extra-hepatic shunts. Extra-heptic shunts are uncommon and are subdivided into those that drain into the superior- or inferior vena cava. The origin of intra-hepatic shunts is mostly congenital and they are found in non-cirrhotic livers (50–76%) incidentally [10]. Acquired causes include cirrhosis and PHTN, liver trauma or rupture of a portal vein aneurysm. Cases of multiple intra-hepatic shunts have been reported that mimic hepatic vascular tumours. Trans-hepatic PSCVs involve intra-hepatic branches of the portal vein that communicate with a systemic vein outside the liver. The most common is the umbilical–paraumbilical shunt from the left portal vein, through the umbilical and paraumbilical venous collaterals, running into the falciform ligament (inferior vein of Sappey), communicating with the inferior epigastric vein and ultimately draining into the inferior vena cava. Other less-common shunts include the right posterior portal branch-to-IVC shunt, bare area of liver shunt and left triangular ligament-type shunt [11]. Among the extra-hepatic shunts, gastrorenal shunts are the most common. They form between the gastric and perigastric varices and the left renal vein, generally through the left inferior phrenic vein that anastomoses with the adrenal vein [12]. Splenorenal shunts are seen as large, tortuous veins in the region of the splenic and left renal hila and drain into an enlarged left renal vein. Splenorenal shunts are sometimes so extremely tortuous that the exact origin of the connection along the splenic vein is difficult to identify. Enlarged splenorenal shunts can clinically lead to encephalopathy. On rare occasions, the spleno-systemic shunt can also occur through the left gonadal vein, leading to a left varicocele in men. Rarer instances of splenorenal shunt in the renal vein are also reported, forming retroperitoneal periureteral varices leading to painless macrohematuria [13].

Dilawari and Chawla have described spontaneous splenoadrenorenal shunts in extrahepatic portal venous obstruction in a series of 20 cases from Chandigarh. They even found that significantly fewer patients had bleeding or oesophageal varices in this group, compared with patients having no spontaneous shunt [14]. Antunez *et al.* reported the presence of a large inferior mesocaval shunt as a rare spontaneous portosystemic shunt in a cirrhosis patient [15]. Gabrael and colleagues described a rare spontaneous portoazygos shunt in a patient with alcoholic cirrhosis and PHTN [16]. The association of a large spontaneous splenorenal shunt with the ‘nutcracker syndrome’ (compression of renal vein) was recently described by Dolowy and colleagues [17]. Culafic *et al.* also reported the presence of spontaneous splenorenal shunt in a patient with liver cirrhosis and hypertrophic caudate lobe [18]. The presence of spontaneous transplenic- and intrasplenic shunts has never before been reported in patients with both cirrhosis and PHTN. Barakat performed a study to describe flow patterns in the portal vascular territory in children with portal vein cavernous deformity. In her study of 12 children, it was found that the intrasplenic veins were dilated in all children with normal flow direction, except in two children who had spontaneous trans-splenic shunts. She pointed out in her conclusion that trans-splenic shunts were uncommon but that their presence is seen in children with cirrhosis and PHTN [19]. The clinical implications of such large shunts are many. It was shown that the presence of large spontaneous shunts in patients with portal hypertension could lead to protracted chronic portosystemic encephalopathy or recurrent episodes of hepatic encephalopathy and that shunt closure in the presence of good hepatic functional reserve resulted in improvement in quality of life. It was also reported that large spontaneous splenorenal shunts were associated with development of hepatocellular carcinoma in the long term, and that the body mass index of the cirrhotic patient predicted the presence of such large shunts [20, 21].

Our case is unique in many respects. Firstly, this report sheds light on the presence of a rare type of shunt (the trans-splenic shunt with intrasplenic collaterals) in an adult patient with compensated cirrhosis. Second, even in the presence of such large shunts, the patient remained relatively stable without any covert or overt features of hepatic encephalopathy. The presence of large splenic arterial aneurysms could be incidental. This could also indirectly relate to the degree of portal pressures that have morphed the portosystemic environment. Nevertheless, this association is novel and further clinical implications need to be investigated in the future. The presence of large shunts in a cirrhosis patient needs to be followed up assiduously. The occurrence of chronic portosystemic or recurrent encephalopathy in such patients warrants closure of the shunt to improve quality of life, and long-term follow-up is advised for early diagnosis of hepatocellular carcinoma.

*Conflict of interest statement:* none declared.
